# Uncovering Consciousness in Unresponsive ICU Patients: Technical, Medical and Ethical Considerations

**DOI:** 10.1186/s13054-019-2370-4

**Published:** 2019-03-09

**Authors:** Benjamin Rohaut, Andrey Eliseyev, Jan Claassen

**Affiliations:** Neurocritical Care, Department of Neurology, Columbia University, New York, NY USA

## Abstract

This article is one of ten reviews selected from the Annual Update in Intensive Care and Emergency Medicine 2019. Other selected articles can be found online at https://www.biomedcentral.com/collections/annualupdate2019. Further information about the Annual Update in Intensive Care and Emergency Medicine is available from http://www.springer.com/series/8901.

## Introduction

Over a decade ago, researchers published the first case of a patient who had been clinically unresponsive for years after traumatic brain injury (TBI) and demonstrated command following using motor imagery paradigms visualized by functional magnetic resonance imaging (fMRI) [1]. The term “cognitive motor dissociation” is gaining popularity to describe this scenario of an inability to behaviorally express preserved cognitive processes [2]. Alternative labels are covert or hidden consciousness and functional locked-in syndrome [3] (see Table 1). A flurry of subsequent studies using fMRI and functional electroencephalogram (fEEG) approaches explored the boundaries of human consciousness following brain injury. This growing body of knowledge is now being discussed in the lay press and is starting to affect clinical medicine, challenging the classical taxonomy of disorders of consciousness ([4] see Table 1). Until very recently, researchers have focused their attention on patients suffering from chronic disorders of consciousness and have generated estimates of cognitive motor dissociation of around 15% using convenience samples of these patients [5]. Detection of cognitive motor dissociation in the acute phase of brain injury may have prognostic significance as these patients are more likely to also recover behavioral command following and have better long-term functional outcomes.

**Table 1:** Definitions of common states of consciousness

	Definition	Other terminologies similar or very close
Behaviorally defined states		
Coma [4]	State of unresponsiveness in which the patient lies with eyes closed and cannot be aroused to respond appropriately to stimuli even with vigorous stimulation (no eye opening or adapted motor response even to painful stimuli).	Coma-1a or 1b^a^ (based on EEG compatibility [1a; e.g., slow unreactive predominant delta] or not [1b; e.g., reactive predominant alpha]). Some authors use a Glasgow coma scale cut-off (e.g., < 8) but this is very misleading since this can include UWS or even MCS patients in whom the ascending reticular activating system (ARAS) is likely to be functional
Unresponsive wakefulness syndrome (UWS) [9]	State of unresponsiveness in which the patient shows spontaneous eye opening without any behavioral evidence of self or environmental awareness	Vegetative state (VS), coma vigil, apallic state, UWS/VS-2a or2b^a^ (CMS excluded [2a] or not [2b] by functional MRI or EEG)
Minimally conscious state (MCS) [8]	State of severely impaired consciousness with minimal but definite behavioral evidence of self or environmental awareness Distinction between MCS “minus” and “plus” has been proposed [3] • MCS–minus: visual fixation/pursuit or adapted motor reaction to pain • MCS-plus: evidence of language processing (e.g., command following, verbalization...)	Cortically Mediated State^a^ (CMS, in that case CMS-3b as based on behavior alone).
Locked-in syndrome (LIS) [4]	State in which the patient is actually conscious but de-efferented, resulting in paralysis of all four limbs and the lower cranial nerves	De-efferented state, Conscious state-4b^a^
Conscious state^b^ [4]	State of full awareness of the self and one’s relationship to the environment, evidenced by verbal or non-verbal (e.g., purposeful motor behavior) behavior	Exit-MCS (or EMCS) when the patient emerged from MCS, Conscious state-4b^a^
Brain functional imaging defined states (e.g., fMRI, fEEG, fNIRS, fPET, fMEG)		
Higher-order cortex motor dissociation (HMD) [26]	Comatose, UWS or MCS-minus (clinically defined) patients that show association cortex responses to language stimuli	CMS-3a^a^
Cognitive motor dissociation (CMD) [2]	Comatose^c^, UWS or MCS-minus clinically defined patients that show MRI or electrophysiologic evidence of command following	Functional locked-in syndrome, Conscious state-4a^a^
Communicating-CMD (Com-CMD)	CMD defined patients able to communicate using a brain computer interface (BCI)	Conscious state-4a^a^

*fMRI* functional magnetic resonance imaging, *fEEG* functional electroencephalography, *fNIRS* function near-infrared spectroscopy, *fPET* functional positron emission tomography, *fMEG* functional magnetoencephalography

^a^Terminology recently proposed by Naccache [50] ranging from 1 to 4 and, taking into account both behavioral (“b”) and brain functional imaging (“a”) evidence. Note that as a consequence, the Cortically Mediated State (CMS) and the Conscious state appear both in the behaviorally and the brain functional imaging sections of this table

^b^Note that as there is no consensus definition of consciousness yet, provided here is a pragmatic operational definition that would match the currently most commonly used definitions. It corresponds to the access consciousness, using the subjective report criterion

^c^The original description actually did not include the comatose state but was included here since the absence of eye opening cannot rule out the possibility of CMD

Few data exist for the early phase after brain injury, such as in the intensive care unit (ICU) setting, when decisions regarding withdrawal of care are more frequently made and prognostic information is needed. Detection of cognitive motor dissociation in the acute setting will face unique challenges including logistics, safety and ethical considerations but also offer great opportunities to affect management. Even though the exploration of consciousness in the acutely brain injured patient is in its infancy, emerging data demonstrating cognitive motor dissociation in patients who are clinically unresponsive raise a number of questions. These questions can be organized around three overarching themes: technical, medical and ethical aspects.

## Technical considerations

### How can we probe consciousness in unresponsive patients?

#### Clinical exam

Intensivists are used to probing the consciousness of brain injured patients during rounds using standard neurological examination techniques. The general principle of the clinical approach for assessment of consciousness is to probe behavior that is non-reflexive and can be considered as intentional. The most common items assessed are reactivity to sound and touch and, if necessary, responsiveness to nociceptive stimuli: is the patient able to open his/her eyes, is he/she attentive or eventually tracking? Clinicians also use simple verbal commands such as “stick out your tongue” and “show me two fingers”. These commands are often combined with a visual cue of the expected response also known as ‘mimicking’. This is employed for patients to minimize the impact of acoustic (i.e., deafness) or speech perception problems (i.e., aphasia). This clinical assessment requires good neurological examination skills to minimize the risk of misinterpretation (e.g., motor responses as part of reflexive responses to pain are thought to be intentional). However, even when performed by experienced clinicians, non-standardized neurological examinations have a high error rate (estimated to be as high as 40% in the chronic setting [6]).

As a part of the neurological exam, many clinicians quantify impairment of consciousness using the Glasgow Coma Scale (GCS). Even though this scale was originally developed to triage acute neurosurgical interventions for TBI patients and to assist prognostication, it may have some utility when applied for this purpose. However, the GCS is a very crude assessment of consciousness, especially when applied to tracheally intubated patients. The Full Outline of Unresponsiveness (FOUR) score may offer an alternative and has been rigorously validated [7]. Since the FOUR does not require verbal responses, it is more applicable for tracheally intubated patients. Probing visual tracking, it also allows a better detection of patients in minimally conscious and locked-in states (Table 2). Currently, the most widely accepted clinical scale designed for assessment of consciousness is the Coma Recovery Scale-Revised (CRS-R) [8]. This comprehensive scale is the gold standard in the field of consciousness research. CRS-R scoring ranges from 0 to 23 according to the presence or the absence of behavioral responses on a set of hierarchically ordered items testing auditory, visual, motor, oromotor, communication and arousal function. State of consciousness is determined by specific key behaviors (and not the total score) probed during the CRS-R assessment. For example, visual pursuit, reproducible movements to command and/or complex motor behavior scores distinguish minimally conscious state from the unresponsive wakefulness syndrome [9], also called vegetative state (see Table 1). However, application of the CRS-R in the ICU can be challenging since assessments of patients may at times require up to 45 min of examination. Another issue is that patients can fluctuate so exams need to be repeated several times before drawing any conclusions. All behavioral scales may incorrectly classify aphasic patients as unconscious but the FOUR score and CRS-R include assessments using visual cues, which may detect signs of awareness in aphasic patients.

**Table 2:**
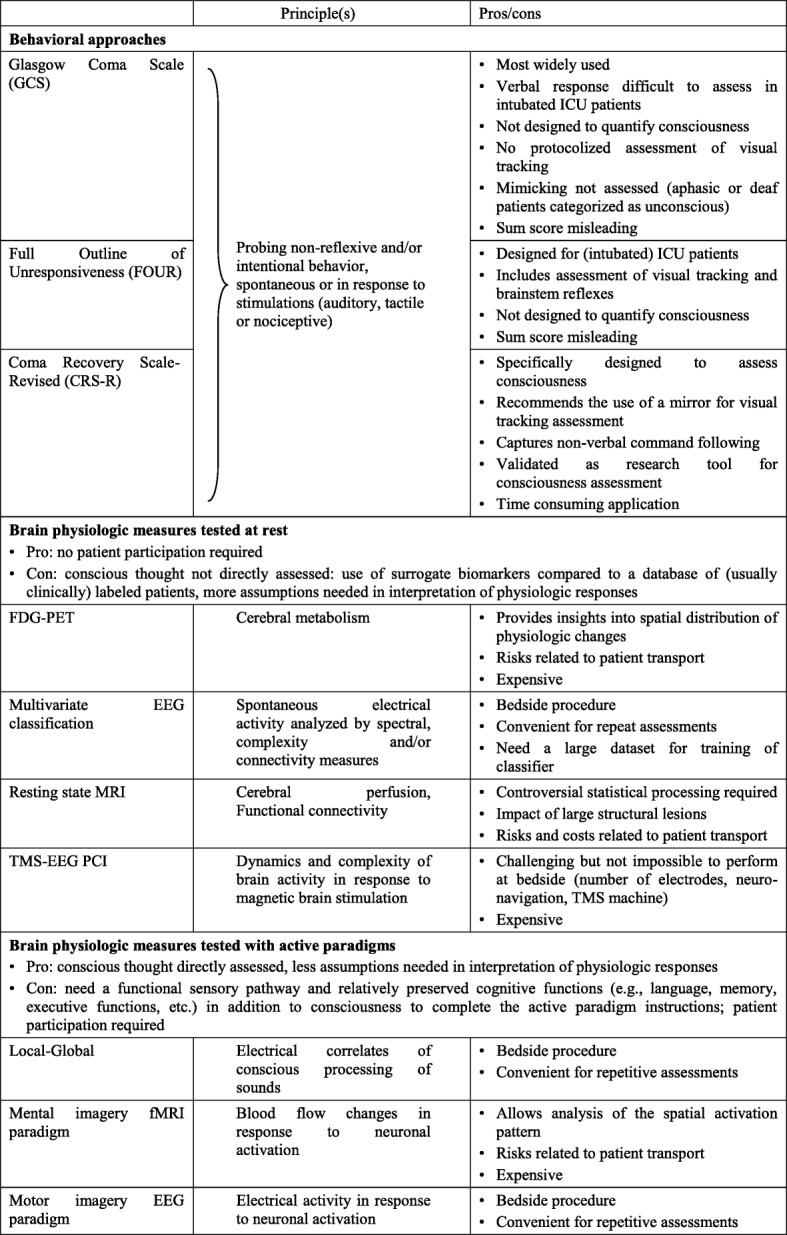
Approaches to assess consciousness

*FDG-PET* fluorodeoxyglucose position emission tomography, *PCI* perturbational complexity index, *TMS* transcranial magnetic stimulation, *EEG* electroencephalography, *(f) MRI* (functional) magnetic resonance imaging, *ICU* intensive care unit

#### Assessing biomarkers that correlate with level of consciousness

The fundamental concept of this approach is based on using neurophysiologic correlates of brain activity as surrogates for levels of consciousness. Such markers include measures of brain metabolism, blood flow and electrical activity. These measures are assessed in a resting condition and correlated with current or future behavioral states. Comparisons are made of the obtained measures in patients appearing unconscious and healthy volunteers. Brain metabolism evaluated using 18F-fluorodeoxyglucose (FDG) position emission tomography (PET)-scans showed that hypometabolism in frontal and parietal cortices is seen in unresponsive wakefulness syndrome [10]. More generally, consciousness seems to vanish when brain metabolism drops below normal activity. Similar approaches have been developed using MRI arterial spin labelling sequences [11].

EEG can, amongst other approaches, be analyzed by decomposing it into spectral patterns, and quantifying complexity and connectivity. Spectral analysis is based on a Fourier transformation and provides information on the power distribution within the respective frequency bands (typically in the δ, θ, α, β and γ bands). Complexity of the EEG can be assessed by entropy measures (e.g., spectral entropy, K complexity, or permutation entropy). Functional connectivity between distant electrodes can be assessed using the spectral dimension (e.g., the debiased weighed phase lag index [wdPLI]) or information theoretical approaches (e.g., the weighed symbolic mutual information [wSMI]) [12, 13]. Similar methodologies assessing cortical functional connectivity have been developed using fMRI [14]. Candidate EEG features can then be used to train on an existing dataset of patients with known level of consciousness using a multivariate classifier to evaluate the EEG of a new patient [15].

Among these techniques, markers derived from resting state EEG seem to be the most promising in the ICU as imaging tests cannot be easily repeated and transport-related risks need to be considered for these sick patients. One study found a correlation between these EEG features (associating spectral, complexity and connectivity measures) and level of consciousness in ICU patients [16]. It is worth noting that the success of any multivariate classification approaches not only relies on the feature’s selection and the quality of the EEG processing but also largely on the quality of the labels provided to the algorithm as a training set. So far, these labels are usually based on behavioral assessments with obvious limitations.

#### Detecting correlates of conscious processing

Another approach to probe consciousness in unresponsive patients derives from neuroscientific and neuropsychological studies that have proposed physiological signatures of conscious processing in response to a given stimulus. One of these techniques focused on a late component of the evoked potential called P300 (it derives its name from the fact that it appears as a positive voltage around 300 ms following a stimulus). One of the most studied paradigms using this phenomenon in consciousness research is called the “Local-Global” paradigm [17]. Schematically this paradigm consists of delivering a subject sequences of sounds that embed two levels of auditory regularity, respectively at a local (within trial) and at a global (across trials) time scale. Whereas detection of local regularity can occur without awareness, detection of global regularity is highly correlated with access consciousness [17] (see [18] for a recent review).

A more recent approach that is at the boundaries between the two approaches described above (i.e., assessing biomarkers that correlate with the level of consciousness as well as detecting correlates of conscious processing itself) consists of measuring the complexity of brain responses to a direct stimulation of the brain using transcranial magnetic (TMS) pulses directly delivered to the parietal cortex. Using a specially designed EEG measure called perturbational complexity index, it is possible to estimate one’s level of consciousness with great accuracy in different settings (i.e., sleep, anesthesia and following brain injury) [19, 20]. This measure summarizes the complexity of the response as well as the functional connectivity in its temporal dynamic dimension. This technique paved the way for alternative interpretations of late evoked brain responses (e.g., the N70) to sensory stimulations observed during the acquisition of somatosensory evoked potentials (SSEP). These brain responses have been associated with prognosis of comatose patients but clinical application has been primarily limited due to a much larger degree of variability when compared to early components of evoked potentials (i.e., the N20 of the SSEP) [21]. Revisiting the neuro-correlates of these late components that follow the classical N20 using new computational measures that quantify connectivity (e.g., wdPLI, wSMI), complexity measures (e.g., permutation entropy) or the perturbational complexity index may provide innovative approaches at the bedside in the ICU to quantify measures that not only correlate with the current state but with future recovery of consciousness.

#### Detecting correlates of command following

Measuring physiologic changes to verbal commands allows the investigator to identify the state of cognitive motor dissociation. This approach has been the first to reliably demonstrate the existence of covert consciousness in patients that clinically meet the criteria of unresponsive wakefulness syndrome [1]. Using fMRI, it is possible to detect whether a patient is able to follow a simple verbal command (e.g., “imagine playing tennis” vs “imagine visiting your home”) by comparing the elicited blood oxygen level dependent (BOLD) imaging signal changes in an unresponsive appearing patient to those seen in a group of healthy volunteers. The first large study suggested that 10% of patients with unresponsive wakefulness syndrome are able to reliably do this [22], a state later termed cognitive motor dissociation [2]. Subsequently, the feasibility of using fEEG paradigms to detect cognitive motor dissociation in unresponsive patients was demonstrated by several teams using a motor imagery EEG paradigm at the bedside [23–27]. Patients with cognitive motor dissociation should be distinguished from patients who are able to process language stimuli (using EEG or fMRI) but without evidence of conscious processing (coined high-order cortex motor dissociation [HMD] [26], see Table 1).

### Pitfalls and caveats of these techniques in the ICU

#### Technical aspects

Limitations of the clinical examination for assessing consciousness have been discussed extensively above. Imaging techniques allow spatial assessments of physical properties of interest (e.g., metabolism, blood flow) but for the purposes of studying ICU patients with impaired consciousness also have limitations in the real world. MRI and PET-scans both require transportation of the patient to the scanner, which potentially exposes patients to multiple risks (e.g., inferior monitoring, non-optimal environment in case of emergency, risk of accidental dislodging of tubes and catheters). To safely acquire MRI scans, sedation and paralytics may be required. Probing for correlates of consciousness under sedation is suboptimal. PET-scans are less problematic since the tracer can be administered just before the scan and the imaging can then be acquired under sedation. However, even for PET scans, transport will be necessary with all of the above outlined risks. Logistical challenges created include the additional personnel required to safely transport patients (e.g., nurse, physician, respiratory technician, MRI technician).

Among all the available techniques, EEG based approaches have enormous advantages in the ICU setting. EEG can be acquired at the bedside within the safe ICU environment. Associated costs for these tests will depend on local reimbursement and healthcare structures. Regardless, imaging tests like MRI or PET scans will likely be much more expensive in most health care systems when compared to EEG or evoked potentials. Additionally, EEG can be repeated many times per day, which is a huge advantage as consciousness is not a static phenomenon but may fluctuate throughout the day (see discussion about clinical limitations above). Assessments before and after interventions, for example, the administration of a medication, are more easily facilitated if repeat testing is easily available. Challenges unique to EEG assessments include electrical noise, which is very prevalent in the ICU environment, and artifacts created by involuntary movements such as myoclonus, respiratory artifact, and coughing. Seizures and other epileptiform patterns are additional major confounders to detect clear signatures of consciousness on the EEG. EEG leads may, in the hectic ICU environment, be removed to allow emergent head CT scans to be obtained to evaluate neurological changes, and placement of EEG leads may be limited by surgical wounds or bandages.

Mental imagery tasks are used both for fMRI and fEEG and are essentially very similar. These probe command following mostly to verbal commands and are fundamentally extensions of the neurological examination. Typically, patients are asked to perform (or to imagine) a motor movement that is thought to elicit a consistent BOLD or EEG signal change to be detected by fMRI or EEG, respectively. Major limitations are that patients need to be able to understand the command (challenging in patients that speak a different language, are deaf or aphasic), are interested in participating (challenging in patients with poor attention, abulia, delirium or in pain), and can keep the command in their working memory long enough to perform the task and to allow the classifier to detect the different brain activities (challenging in patients with advanced dementia). Importantly, both with fMRI and fEEG, patients can be identified that are conscious but for the reasons outlined above, consciousness cannot be ruled out for any of the patients that are classified as unconscious.

#### Confounding factors

Among the major confounding factors for the assessment of consciousness in the ICU setting, the following take a central role: sedation and delirium. Determining the exact level of consciousness is usually not a major concern for the clinician treating deeply sedated patients (e.g., those receiving treatment of refractory status epilepticus or intracranial hypertension). Medications used in this context include those used for general anesthesia. On the other hand, in patients who receive lower doses of sedation, assessments of consciousness can be very challenging. However, pharmacodynamics and pharmacokinetics are altered in deeply sedated patients in the ICU receiving prolonged courses of sedatives. Inferring absence of consciousness from the absence of responsiveness may lead to the erroneous assumption of unconsciousness in sedated patients as recently demonstrated in a study revealing that conscious experiences may occur under propofol-induced unresponsiveness [28].

Another caveat is the high prevalence (30%) of delirium in the ICU [29]. Although it is relatively easy to diagnose the classical form of delirium and to demonstrate consciousness (even though in an altered form), the commonly coined ‘hypoactive delirium’ that can represent half of the delirium can be more difficult to identify [30]. Delirious patients usually have attention deficit that could hamper the focus required for the detection of signature of conscious processing of a stimulus (e.g., the Local-Global paradigm) or the sustained attempt to follow verbal commands.

### What are we detecting exactly?

Applying the above introduced techniques, three different kinds of signals as surrogates of covert consciousness can be detected in an unresponsive ICU patient: (1) a physiological biomarker that correlates with level of consciousness at the group level (e.g., FDG-PET scan measure of global brain metabolism or multivariate analysis of EEG features [perturbational complexity index]); (2) a correlate of conscious processing of a given stimulus at the individual level (e.g., the P3b using the Local-Global paradigm, perturbational complexity index); and (3) appropriate and sustainable brain activities in response to verbal commands at the individual level (e.g., using the motor imagery EEG paradigm). We propose that the last category represents at this point the most direct and convincing evidence of covert consciousness that has been termed cognitive motor dissociation. Indeed, physiologic biomarkers that correlate with consciousness have been developed on models trained on large datasets using clinical labels. Consequently, the confidence of how accurately a given patient is classified using these approaches mainly relies on the quality of the labels used in the training set. These labels are derived from clinical examinations which we know are imprecise. The relevance of neural correlates of conscious processing such as the P3b are still debated. In addition, evidence of conscious processing of simple stimuli does not imply the existence of conscious processing of more complex mental contents. Following commands revealed by fMRI or fEEG appears as the strongest evidence since it usually relies on statistics performed at the patient level (using for instance machine learning and permutation test).

## Medical considerations

### Prognostication and medical decision making

Prognostication in the acutely unconscious patient is one of the most challenging problems that intensivists face when taking care of brain injured patients. Determining goals of care is paramount in a setting where survival can mostly be provided but may not be desirable if the quality of survival is clearly against the patient’s pre-stated wishes. Actual and predicted recovery of consciousness are major factors that physicians and families take into account when deciding about goals of care. Prognostication is frequently inaccurate but clinicians usually take into account the clinical examination, structural neuroimaging, biomarkers and electrophysiological testing. In addition, they need to consider the dynamic nature of the brain injury as well as potential confounding factors, such as sedation, metabolic derangement, and or mental disturbance (e.g., delirium). The premorbid condition and age of the patient play a role for most conditions. Disease specific prognosis markers can help predict long-term functional outcome (usually 6–12 months) [31] but uncertainty usually remains and clinicians should be aware of the risks of ‘flawed reasoning’ given the high degree of complexity that may occur as a result of cognitive biases [32].

Caution is warranted therefore against any studies that further add to the degree of complexity in assessing these patients. However, the existence of cognitive motor dissociation during the acute stage of brain injury, if confirmed, will likely dramatically change the assessment of prognostication of these patients. The recently published guidelines by the American Academy of Neurology for the management of patients suffering from chronic disorders of consciousness may serve as an indicator for what may occur for acutely brain injured patients. These guidelines underline the possibility of improvement for unresponsive patients months following acute brain injury and, accordingly, urge to replace the term ‘permanent’ vegetative state by ‘chronic’ vegetative state (or unresponsive wakefulness syndrome) [6]. The acknowledgement of this high degree of uncertainty at the subacute stage of acute brain injury, and usually with fewer data than in the chronic setting, should be remembered when elaborating a poor prognosis based on limited data a few days after acute brain injury.

### Pain management

Considering that 15–40% [5, 26, 27] of unresponsive patients might be actually conscious and able to experience pain without any way to express suffering, caregivers need to consider pain medications whenever a medical condition is bound to generate nociceptive inputs. Invasive procedures in unconscious appearing patients should be performed using the same analgo-sedative management approach as in an awake, communicative patients.

### Recovery of communication abilities

A common challenge for patients in the ICU is their inability to consistently and effectively communicate their most fundamental physical needs [33]. Conscious patients in the ICU commonly suffer from unrecognized pain, discomfort, feelings of loss of control and insecurity, depersonalization, anxiety, sleep disturbances, fear and frustration [34]. The primary means of communication for these patients is the use of non-vocal techniques, such as lip reading and gestures; however these methods are often inadequate for effective communication [35]. In addition, the recent description of cognitive motor dissociation during the acute phase of brain injury increases the potential number of patients in a situation of inefficient communication [26].

**Fig. 1:**
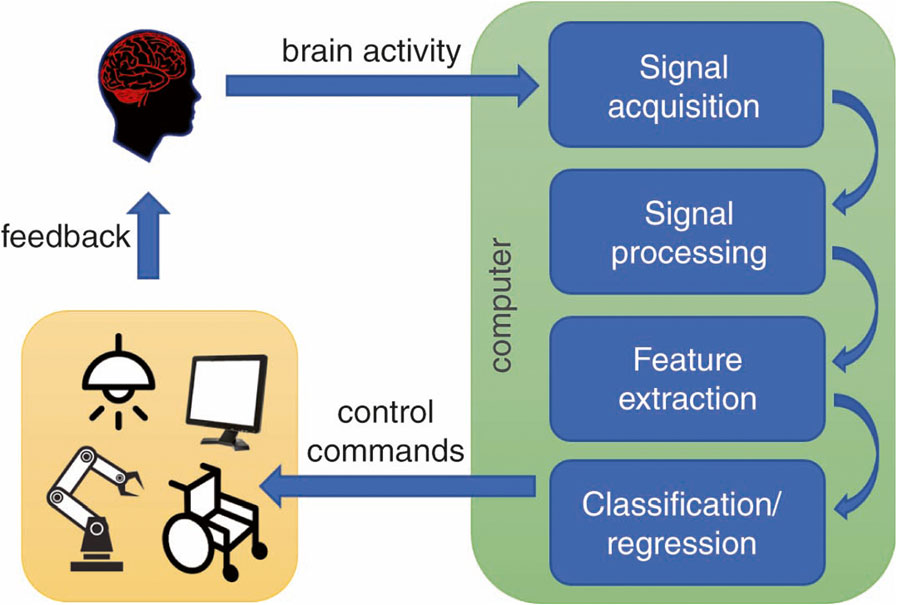
Brain-computer interface systems. Brain-computer interface systems use state-of-the-art machine learning methods to decode brain activity. A brain-computer interface system is realized using several components: (1) brain signal activity acquisition: electroencephalogram (EEG), electrocorticography (ECoG), functional magnetic resonance imaging (fMRI), functional near-infrared spectroscopy (fNIRS), etc.; (2) signal processing: band-pass filtering, outlier removal, artifact correction, normalization, etc.; (3) feature extraction: gain task-relevant information from acquired data; (4) classification/regression: decode the intended action of the subject by applying machine learning methods; (5) control commands to external devices: screen, wheelchair, exoskeleton, etc.; (6) feedback: the subject receives feedback about how well he/she performed in a certain training task

Brain-computer interface (BCI) systems have recently generated interest as a method to facilitate contact with the ICU patients. BCIs translate the patient’s cerebral electrical activity, typically recorded by EEG, into computer commands bypassing other body functions (Fig. 1). Although a variety of BCI systems have been proposed for rehabilitation purposes [36, 37], the number of BCIs, assisting with communication of the typical physical and emotional needs of the critically ill, remains significantly limited [38, 39].

The main restriction for practical application of BCI systems in the ICU is lack of reliability due to the considerable number of distractions, possible extinction of goal-directed thinking, deterioration of patient attention control, etc. Another specific challenge includes eyelid apraxia or other visual impairments that preclude the use of classical visual cues. In addition, owing to extended bedrest and skin breakdown, and pain medication, the tactile input channel is also sometimes impaired. Auditory cues allow only very limited information to be transferred, like simple questions or commands. Moreover, many training sessions could be required to teach patients to use BCI technology which will be challenging with distracted patients in pain and evolving medical conditions. Therefore, current BCIs in the ICU focus on quick and reliable signaling (e.g., ‘yes’/‘no’ binary signals [38, 39] or steady-state visual evoked potential (SSVEP) based communication [38, 39]) rather than spelling of words or sentences.

Despite the limitations, BCI technology has the potential to significantly increase patient autonomy allowing more efficient pain management as well as better interaction with the external environment (e.g., bed position, call button, lights, television, etc.). Portable EEG-based BCI has been used in one study for the detection of consciousness [40]. It uses Pavlovian semantic conditioning to discriminate between cognitive ‘yes’ and ‘no’ responses. However, it demonstrated reliable level of performance only for offline classifiers in one out of three locked-in state patients.

To improve the reliability of BCI systems, utilization of hybrid BCIs, combining either sequential or simultaneous integration of different data sources, has been proposed [41]. In hybrid BCIs, EEG data could be complemented with other brain as well as non-brain modalities, such as functional near infrared spectroscopy (fNIRS), electrooculography (EOG), and electromyography (EMG), heart rate, hemodynamic response, etc. [42]. Due to their advanced reliability, hybrid BCIs could be especially efficient for ICU application.

Despite the drawbacks of current BCI systems, their main advantage is the potential for instant data processing. Moreover, computationally efficient methods proposed for robust treatment and adaptive modeling of complex data streams in real-time [43], allow implementation of BCIs into a personal computer or even tablet for easy installation in the ICU. Fast feedback of the real-time BCI systems simplifies the training process for patients and enables the medical staff to respond more rapidly to the time-sensitive needs of the patients.

The proportion of patients with cognitive motor dissociation who would be able to use a BCI to communicate (that could be called “communicating cognitive motor dissociation” (Table 1)) remains to be determined. According to the obstacles described above, we can hypothesize that only cognitive motor dissociation patients with preserved high cognition capacities (language, memory, executive function, etc.) will be able to use a BCI. However, it is worth noting that these devices would also benefit a larger disabled patient population, such as lock-in syndrome, hemiplegic or paraplegic patients.

## Ethical considerations

Currently, the majority of brain injured patients who die in the ICU do so as a direct consequence of withdrawal of life-sustaining therapies [44]. Lack of consciousness has a major impact on medical decision making, particularly withdrawal of life-sustaining therapies. Indeed, many prognostication tools (e.g., following cardiac arrest, intracranial hemorrhage, subarachnoid hemorrhage) attribute a huge weight on the level of consciousness (usually crudely assessed by the GCS). From that perspective, the use of the most accurate technique to detect consciousness and capture cognitive motor dissociation needs to be a major focus of our efforts. Consciousness is an irreducible component of personhood and a central tenet of the Belmont Report [45].

Decisions to withdraw life-sustaining therapies are frequently made within the first weeks following brain injury, frequently within hours or days of the injury. Increasingly, however, studies show that delayed recovery is more common than previously thought. Consciousness is not systematically quantified to improve prognostic tools. Concerns relative to these failures of our moral obligations to probe residual consciousness and to elaborate a prognosis as precise as possible have been raised to reduce our ‘neglect’ [46]. In that context, recent recommendations by an association of British critical care medical societies to (1) extend the observation time window; (2) use multiple exploration techniques; and (3) consider the involvement of a “neuroscience team” are a step in the right direction [31]. These recommendations should be put to test in cost-effectiveness studies and undergo open public debate. Caregivers are torn between on the one hand providing the highest level of care (which includes providing sufficient time to achieve a reliable prognosis) and on the other hand to guarantee equity by providing as many patients as possible access to the scarce resource of a highly specialized critical care unit (constraints of limited resources). The question of how much money a given society is willing to invest in order to maximize chances of recovery should be openly discussed considering the number of patients that could benefit from this service. Societal burden of potential long-term survival with disability also needs to be considered. To date these considerations are unfortunately handled locally by caregivers and are at the roots of a great variability in practice and differences in provided level of care.

### Ethical aspects raised by covert consciousness in the ICU

The potential existence of covert consciousness in unresponsive patients in the ICU raises many concerns. For example, considering the pain management discussed above, one might want to preserve any suffering or pain and administer sedatives and pain killers whenever a doubt exists [45]. On the other hand, one might want to preserve covert consciousness in order to maximize the chances of detection to support prognostication and possibly provide a communication channel with these patients in the future. This dilemma has been known for years by clinicians assessing consciousness using fMRI of EEG on a regular basis.

## Conclusion

Forty-five years ago, Jennet and Plum acknowledged that the absence of behavioral evidence of awareness could erroneously suggest the absence of consciousness (“it seems that there is wakefulness without awareness”), stating that there is no “reliable alternative available to the doctor at the bedside, which is where decisions have to be made” [47]. This visionary prediction is now reality. Given the available data it is clear that behavioral criteria alone are not sufficient to accurately define consciousness states and it is no wonder that recent discoveries on consciousness disorders have led to revisiting of the taxonomy of patients with disorders of consciousness [48–50]. For example, recent debate has emphasized the lack of homogeneity of the minimally conscious state (minimally conscious state minus/plus dichotomy [3]) category and even challenged assumptions of the nature of consciousness in minimally conscious state (proposing to replace this term by cortically mediated state, to avoid any inference from a patient’s subjectivity) [50]. Increasing diagnostic precision is bound to increase prognostic accuracy and will hopefully lead to tailored therapeutic interventions. The intensivist needs to stay in tune with this rapidly evolving area that tackles some of the most complex neuroscientific concepts (i.e., consciousness, neuro-prognosis, neuro-repair), cutting edge technologies (advanced brain imagery and signal processing) and fundamental ethical questions (autonomy, equity, quality of life and life or death decisions).
